# Supervised and Non-Supervised Exercise Programs for the Management of Cancer-Related Fatigue in Women with Breast Cancer: A Systematic Review and Meta-Analysis

**DOI:** 10.3390/cancers14143428

**Published:** 2022-07-14

**Authors:** Gonzalo Reverte-Pagola, Horacio Sánchez-Trigo, John Saxton, Borja Sañudo

**Affiliations:** 1 Department of Physical Education and Sport, Universidad de Sevilla, 41012 Sevilla, Spain; fstrigo@us.es (H.S.-T.); bsancor@us.es (B.S.); 2 Department of Sport, Health and Exercise Science, Faculty of Health Sciences, University of Hull, Hull HU6 7RX, UK; john.saxton@hull.ac.uk

**Keywords:** breast, neoplasms, fatigue, exercise, COVID-19

## Abstract

**Simple Summary:**

Physical exercise is considered to be a non-pharmacological strategy for reducing symptoms of cancer-related fatigue (CRF) in women with breast cancer (BC). The aim of this systematic review and meta-analysis is to assess the effects of non-supervised exercise programs by comparison with the effects of supervised exercise interventions for CRF in this patient group. Randomized controlled trials that investigated the effect of exercise on CRF in women during or after adjuvant BC treatments were searched for using PubMed, SportDiscus, Web of Science, CINAHL, PsycInfo, CENTRAL, ClinicalTrials.gov and EMBASE until 29 June 2022. Thirty-one studies met the inclusion criteria (*n* = 2964). Both non-supervised and supervised exercise programs significantly reduced CRF. There were no significant differences between non-supervised and supervised exercise programs according to random effects analysis. However, in the short term, supervised training programs may have a greater effect. In contrast, long-term differences in CRF between supervised and non-supervised exercise programs are not apparent.

**Abstract:**

Physical exercise is considered to be a non-pharmacological strategy for reducing symptoms of cancer-related fatigue (CRF) in women with breast cancer (BC). This systematic review and meta-analysis aims to assess the effects of non-supervised exercise programs in comparison with the effects of supervised exercise interventions for CRF in BC patients. Randomized controlled trials that investigated the effect of exercise on CRF in women were searched for until 29 June 2022. Inclusion criteria comprised women diagnosed with BC; exercise-based interventions; trials comparing at least one exercise group vs. a control group; trials that assessed exercise effects on CRF. Thirty-one studies met the inclusion criteria (*n* = 2964). Both non-supervised and supervised exercise programs significantly reduced CRF (standard mean difference (SMD) = −0.46, confidence interval (CI) = (−0.64, −0.28), *p* < 0.0001 and SMD = −0.74, CI = (−0.99, −0.48), *p* < 0.0001, respectively), without statistical difference (*p* = 0.09). However, a short-term training program subgroup analyses showed significant differences between supervised and non-supervised training programs (*p* = 0.01), showing that supervised training programs have a greater effect (SMD = −1.33, CI = (−1.92, −0.73), *p* < 0.0001) than non-supervised ones (SMD = −0.44, CI = (−0.78, −0.11), *p* = 0.009). Both supervised and non-supervised exercise programs may reduce CRF in BC patients; however, in the short-term, supervised exercise may have a greater effect on CRF in BC patients.

## 1. Introduction

Breast cancer (BC) is the most frequently diagnosed cancer in the vast majority of countries and is also the leading cause of cancer death in over 100 countries [1]. Even though BC mortality rates are decreasing in most high-income countries, BC incidence rates are increasing [2] and the costs associated with this condition are rising [3]. It is estimated that cancer caused almost 10 million deaths in 2020, and 19.3 million new cases were diagnosed [4]. Globally, female BC has surpassed lung cancer as the most diagnosed cancer with 2.3 million new cases annually, and with a 6.9% mortality rate each year [4]. In Europe, the economic costs associated with BC have reached EUR 15 billion per annum [5] and in the USA, the total national cost for medical services and oral prescription drugs for BC was USD 26 billion in 2015 [3].

Disease management in this population is multimodal. Radiotherapy after breast-conserving surgery is associated with a 21.7% reduction in 10-year local recurrence, a 5.4% reduction in 15-year BC mortality, and a 5.3% reduction in 15-year overall mortality [6]. Chemotherapy is also used as an adjuvant treatment for preventing recurrence in many patients at stage I–III, despite the associated short- and long-terms risks [7]. However, these treatment options have many adverse side effects, including fatigue, osteoporosis, infections, cardiotoxicity, cognitive deficits, sleep disturbances, anemia, sexual problems, hot flashes, and pain [8,9,10,11]. Cancer-related fatigue (CRF) is one of the most common and debilitating side effects that can persist for years after treatment [12]. This symptom adversely affects quality of life and may be a risk factor for reduced survival [12].

Current evidence suggests that non-pharmacological strategies such as exercise are more effective for ameliorating CRF than pharmacological interventions [13]. Systematic reviews have assessed the efficacy of different types of exercise for reducing CRF in this population, with studies comparing the relative impacts of aerobic, resistance and multi-modal programs [14,15]. Despite the promising evidence reported in these studies, patients are often reluctant to engage in structured exercise programs because of a belief that treatment-related side effects (e.g., fatigue, nausea, pain, and shoulder stiffness) could be exacerbated [16]. Misguided risk perceptions and safety concerns are commonly reported barriers to physical activity in this population, which could be attributed to vague, inconsistent, or a lack of credible information on physical activity being provided by healthcare professionals in cancer care settings [16].

Furthermore, the COVID-19 pandemic has negatively affected BC patients’ lifestyle behaviors [17] and hampered the clinical management of this population [18], as well as made it difficult for patients to benefit from their social support structures [19]. Social distancing measures have meant that many BC survivors have decreased their physical activity and have adopted a more sedentary lifestyle [20,21]. In fact, recent evidence suggests that social distancing and social isolation policies have created a “sedentaryogenic” environment, in which meeting physical activity guidelines has become especially challenging for people with cancer [22]. This has raised the question of whether home-based exercise prescription, perhaps with remote support, can positively impact CRF and other common side effects of treatment in BC patients [17]. On the other hand, recent studies have underlined the importance of supervised exercise in this context [23].

Thus, this systematic review and meta-analysis aims to determine the efficacy of non-supervised and supervised exercise programs for ameliorating symptoms of CRF in women who have been treated for BC, and to evaluate which characteristics optimize the effectiveness of non-supervised exercise programs to reduce CRF in this population.

## 2. Materials and Methods

### 2.1. Protocol and Registration

The protocol of this review is registered in the Prospective Register of Systematic Reviews (PROSPERO) with registration number: CRD42021240887. Reporting was guided by the standards of the Preferred Reporting Items for Systematic Review and Meta-Analysis (PRISMA) Statement [24]. Further details (PRISMA checklist) can be found in the Supplemental Material (File S1).

### 2.2. Search Strategy

Two authors (GRP and HST) independently searched PubMed, SportDiscus, Web of Science, CINAHL, PsycInfo, Cochrane Central Register of Controlled Trials (CENTRAL), ClinicalTrials.gov, and EMBASE for articles published from inception until 29 June 2022. These electronic databases were searched systematically with a Boolean search strategy comprising population (i.e., adult), condition (i.e., breast cancer), intervention (i.e., exercise), and outcome (i.e., fatigue). Further details (i.e., the search strategy) can be found in Appendix A. All citations were entered into a reference management software program (Mendeley Desktop Software, version 1.19.4, Elsevier, Amsterdam, The Netherlands). Duplicates were excluded automatically, and the remaining studies were assessed according to the eligibility criteria.

### 2.3. Eligibility Criteria

Only studies that met the following eligibility criteria were included in the meta-analysis. The PICOS (patient population, intervention, comparative controls, outcomes, study type) framework was applied to formulate eligibility criteria and ensure scientific thoroughness [25].

#### 2.3.1. Population

Adult women (>18 years old) diagnosed with metastatic or non-metastatic BC that have completed or are undergoing BC treatments. 

#### 2.3.2. Intervention

Interventions were based on exercise, defined as any planned, structured, and repetitive bodily movement completed to improve or maintain one or more components of physical fitness [26]. Multi-component interventions that consisted of exercise with, for example, physiotherapy or educational sessions were also considered. However, interventions that consisted of exercise plus diet were excluded. Studies that only compared exercise with another pharmacological or non-pharmacological treatment (e.g., diet), were also excluded.

#### 2.3.3. Comparison

For the eligibility criteria regarding the comparative control groups, studies were only included if they had a non-intervention control group with no changes in habitual activity levels, for example, educational groups, self-shoulder stretching exercises, the maintenance of a sedentary lifestyle, muscle relaxation, or oncologist verbal recommendations.

#### 2.3.4. Outcome

Studies that assessed exercise effects on CRF were measured subjectively. The National Comprehensive Cancer Network (NCCN) [27] defines CRF as “a distressing, persistent, subjective sense of physical, emotional, and/or cognitive tiredness or exhaustion related to cancer or cancer treatment that is not proportional to recent activity and interferes with usual functioning”.

#### 2.3.5. Study Design

Only randomized controlled trials (RCT) were included.

### 2.4. Study Selection and Data Extraction 

Study selection was performed independently by GRP and HST. First, titles and abstracts were screened to exclude irrelevant studies. Then, full-text articles were evaluated to apply inclusion criteria. Disagreements were solved in a consensus meeting with a third reviewer (BSC). Finally, the key data (age, sample, level of adherence, duration of the programs, supervision or non-supervision, typology of the exercise intervention, and instrument used for the assessment of CRF) were collected from each selected study. Pre- and post-test results and standard deviations (SD) were extracted from the intervention and control groups of each paper. If any data were unavailable from the papers, authors were contacted. If a paper reported using questionnaires to measure CRF but did not report this outcome, the authors were contacted for further information to avoid publication bias. No restrictions were applied for publication date. 

### 2.5. Risk of Bias

Two review authors (GRP and HST) independently assessed the risk of bias using the Cochrane “risk of bias assessment tool” [28]. Risk of bias was assessed with the following domains:

- Random sequence generation (selection bias);
- Allocation concealment (selection bias);
- The blinding of participants and personnel (performance bias);
- The blinding of outcome assessment (detection bias);
- Incomplete outcome data (attrition bias);
- Selective reporting (reporting bias);
- Other bias.

Each domain was judged as “low risk of bias” if requirements were adequately fulfilled as described by Higgins, 2011; as “high risk of bias” if requirements were not adequately fulfilled; or as “unclear risk of bias” if data provided were insufficient for a judgement [28]. Scores were based on the information available from the published versions and from communication with the authors. Studies scoring less than 4 were considered low quality due to high risk of bias and, therefore, were not considered in the present review. Funnel plots were examined to assess publication bias. If the funnel plot showed symmetry, publication bias was ruled out. 

### 2.6. Statistical Analysis

Meta-analyses were performed using Review Manager V.5.4. (Cochrane Collaboration, Copenhagen, Denmark). Since CRF was assessed with a diverse range of questionnaires, size effect was measured as the standardized mean difference (***SMD***) between the experimental and control group, computed as

(1) $$SMD=\frac{mean(D_{Int})-mean\left(D_{Ctr}\right)}{SD\left(D_{Int,Ctr}\right)}$$

where ***D_Int_*** and ***D_Ctr_*** are the post–pre differences in the intervention and control groups, respectively, and ***D_Int,Ctr_*** is the post–pre difference in the combined group [29]. When the ***SD*** of the post–pre differences were not reported, it was calculated from the confidence interval (CI), standard error, or *p*-value of the absolute change of CRF using standardized formulae [30]. If none of these data were available, the following formula was employed:

(2) $$SD=\sqrt{SD_{pre}^{2}+SD_{post}^{2}-\left(2\cdot r\cdot SD_{pre}\cdot SD_{post}\right)}$$

where ***r*** is the correlation coefficient that describes how similar the pre- and post- measurements were across participants [30]. The r value in the intervention and control groups was estimated at 0.8 after averaging it for those studies that reported full data. Replacing ***r*** with 0.7 in sensitivity analyses did not affect the findings of this study.

***SMD*** were considered statistically significant at the 5% level (*p* < 0.05), and classified as small (0.1–0.3), medium (0.3–0.6) or large (>0.6) [31]. In studies with two intervention groups and a single control group, the sample size of the control group was halved in the statistical analysis to avoid miscalculating the population size [32]. SMD were calculated with a random effects model, along with 95% confidence intervals (CI). Heterogeneity was measured using the I^2^ statistic [33], with I^2^ values of 25%, 50% and 75% being considered low, moderate, and high [34]. 

Subgroup analyses for exercise program duration and adherence to the programs were conducted comparing supervised vs. non-supervised interventions. 

## 3. Results

### 3.1. Characteristics of Included Studies

**Figure 1:**
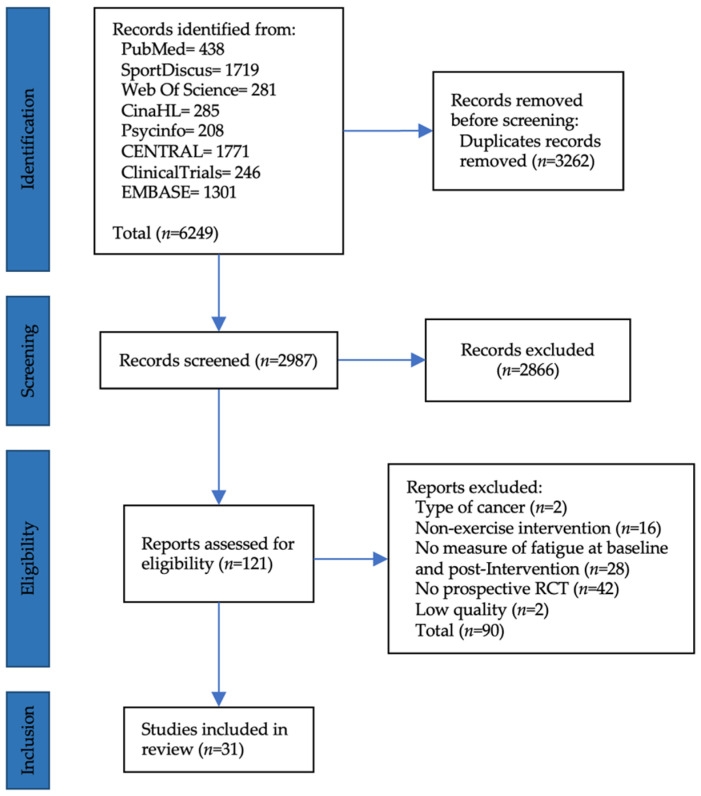
Flowchart for search strategy methods. Flowchart is performed according to PRISMA framework [24].

The initial search yielded 5846 studies (Figure 1). After the removal of duplicated records, 2987 studies were screened based on titles and abstracts. A total of 2866 articles were excluded because they were not related to the topic of the present review and the number of full-text studies evaluated for inclusion was 121. Figure 1 shows the flow of studies and the reasons for study exclusion. Two studies were excluded because participants were not BC patients, sixteen studies used non-exercise-based interventions, twenty-eight studies did not measure fatigue at baseline or after the intervention and forty-two were not prospective RCTs. Two studies had low methodological quality. In total, 31 studies (*n* = 2964) met the inclusion criteria and were included in the analysis (Figure 1). 

Of the 31 included studies, 9 originated from USA [35,36,37,38,39,40,41,42,43]; 2 from Spain [44,45]; 3 from Canada [46,47]; 2 from Australia [48,49]; 2 from Netherlands [50,51]; 2 from Germany [52,53]; 2 from Taiwan [54,55]; 2 from the UK [56,57]; 1 from Italy [58]; 1 from Turkey [59]; 1 from Norway [60]; 1 from South Korea [61]; 1 from Thailand [62]; 1 from Iran [63] and 1 from Sweden [64].

Table S1 shows that, of the non-supervised included studies, 10 interventions were exclusively endurance exercise [36,37,40,41,42,54,57,59,62,65]; 4 combined endurance with resistance exercise [45,49,51,60]; and 2 studies involved other types of exercise such as Nia exercise [38] and yoga [39].

Of the supervised included studies, six interventions were exclusively endurance exercise [43,46,47,56,63,64]; four interventions were exclusively resistance exercise [46,48,52,53]; six combined endurance with resistance exercise [50,51,58,59,61,64] and three studies involved other types of exercise such as yoga [35,55] and hydrotherapy [44].

The studies included a total of 2964 women, 1629 who participated in supervised and 1335 who participated in non-supervised exercise programs. Sample sizes ranged from 14 to 377, with a median of 91 participants. All studies included one experimental and one control group, but some studies included one extra intervention group [46,49,51,59,64]. The duration of the exercise programs ranged from 4 to 32 weeks and the frequency of exercise ranged from two to seven weekly training sessions of 10 to 90 min in length.

### 3.2. Participants

Only studies which included adult women (>18 years old) diagnosed with metastatic or non-metastatic BC and women who had completed or were undergoing BC treatments were selected. As shown in Table S1, the age range was 43–63 years and the mean age of the participants was 51.7 years. Almost half the sample (46%) had completed BC treatment when they started the exercise intervention and 54% of the sample was receiving BC treatment (i.e., chemotherapy, radiotherapy) during the exercise program.

### 3.3. CRF Assessment

The questionnaires used to assess CRF in the included studies were: the Brief Fatigue Inventory [66], the European Organisation for Research and Treatment of Cancer [67], the Functional Assessment of Cancer Therapy—Fatigue [68], the Fatigue Assessment Questionnaire [69], the Multidimensional Fatigue Inventory [70], the Piper Fatigue Scale [71], the Profile of Mood State [72], the Schwartz Cancer Fatigue Scale [73], a quality of life breast-cancer-specific questionnaire [74], and visual analogue scales [75,76]. All the questionnaires assessed CRF subjectively and most of them used a Likert scale of 5 or 10 points to indicate the severity of the symptom.

### 3.4. Risk of Bias

**Figure 2:**
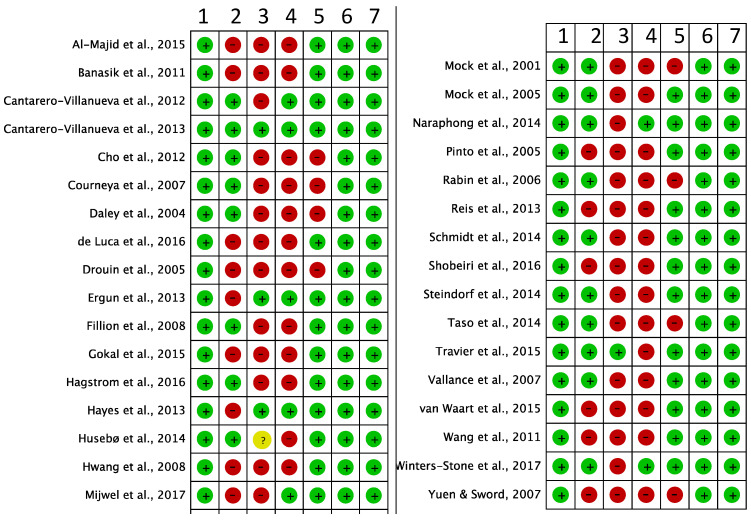
Methodological quality of included studies using the Cochrane “risk of bias assessment tool” [35,36,37,38,39,40,41,42,43,44,45,46,47,48,49,50,51,52,53,54,55,56,57,58,59,60,61,62,63,64,65,77,78]. 1: Random sequence generation (selection bias). 2: Allocation concealment (selection bias). 3: Blinding of participants and personnel (performance bias). 4: Blinding of outcome assessment (detection bias). 5: Incomplete outcome data (attrition bias). 6: Selective reporting (reporting bias). 7: Other bias. Green: Low risk. Red: High risk. Yellow: Unclear risk.

As reported in Figure 2, two studies were considered to have a high risk of bias (score < 4) and, consequently, both were excluded from the analysis [77,78]. All studies that were meta-analyzed were considered to be “high quality” with a score ≥ 4.

#### Publication Bias

**Figure 3:**
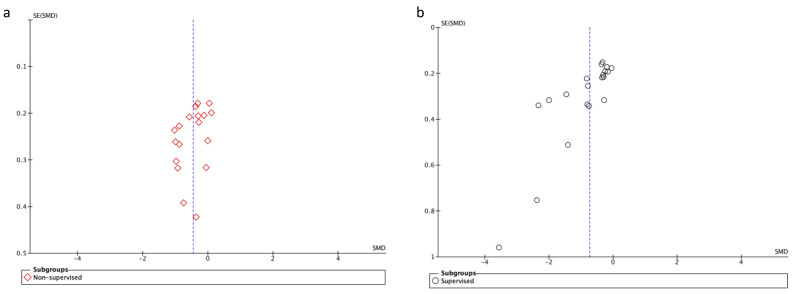
(**a**) Funnel plot of standard error (SE) against standardized mean difference (SMD) for the assessment of publication bias in the investigation of the CRF outcome in non-supervised training programs. (**b**) Funnel plot of standard error (SE) against standardized mean difference (SMD) for the assessment of publication bias in the investigation of the CRF outcome in supervised training programs.

As shown in Figure 3, the funnel plots show asymmetry, indicating the presence of publication bias for the CRF outcome in non-supervised and supervised exercise studies

### 3.5. Pooled Effects

**Figure 4:**
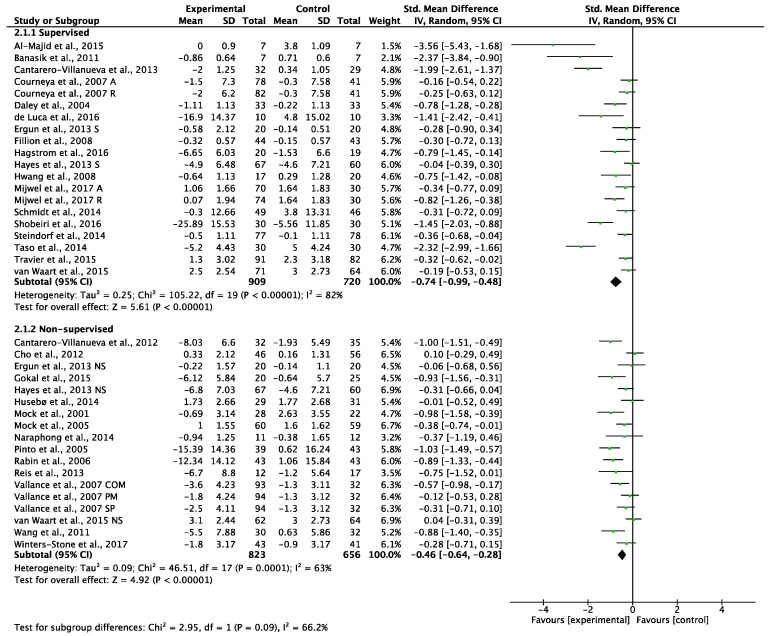
Meta-analyses of the effects of supervised [35,43,44,46,47,48,49,50,51,52,53,55,56,58,59,61,63,64] and non-supervised exercise [36,37,38,39,40,41,42,45,49,51,54,57,59,60,62,65] in CRF. A: aerobic exercise group; COM: combination of printed materials and step pedometer; NS: non-supervised exercise group; PM: printed materials; R: resistance exercise group; S: supervised group; SP: step pedometer.

The pooled results showed a significant reduction in CRF in favor of intervention groups receiving supervised exercise programs (SMD −0.74, 95% CI −0.99 to −0.48, (*p* < 0.00001), I^2^ = 82%; Figure 4). Non-supervised exercise programs also resulted in a significant reduction in CRF versus non-exercise controls (SMD = −0.46, 95% CI = −0.64 to −0.28, (*p* < 0.00001), I^2^ = 63%; Figure 4). The test for the subgroup differences demonstrated non-significant differences between supervised and non-supervised exercise programs in the management of CRF in BC patients (*p* = 0.09) (Figure 4). A sensitivity analysis omitting each study was performed and did not affect the findings.

#### 3.5.1. Subgroup Analysis: Duration of the Training Programs

A subgroup analysis was performed to assess the effect of exercise program duration on CRF. Short-term (≤12 weeks) and long-term interventions (>12 weeks) were compared for both supervised and non-supervised programs.

##### Short-Term Interventions (Less Than 12 Weeks)

**Figure 5:**
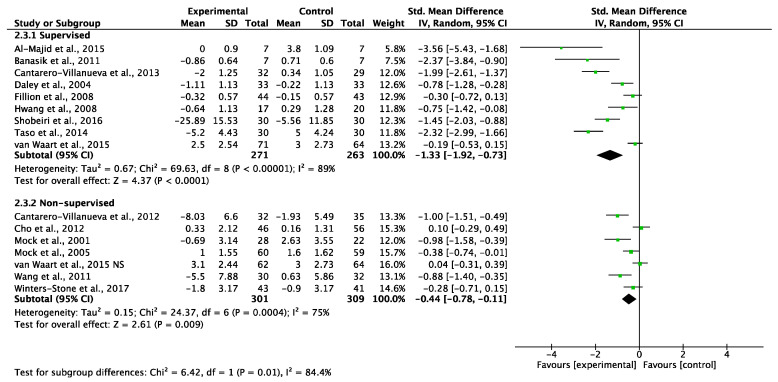
Meta-analyses of the effects of supervised [35,43,44,47,51,55,56,61,63] and non-supervised [36,37,39,41,45,51,54] exercise interventions of less than 12 weeks on CRF in BC patients. NS: non-supervised exercise group.

Short-term supervised exercise programs showed a significant reduction in CRF (SMD: −1.33, 95% CI −1.92 to −0.73, *p* < 0.0001; Figure 5) with high statistical heterogeneity (I^2^ = 89%). Non-supervised exercise programs also showed a significant reduction in CRF (SMD: −0.44, 95% CI −0.78 to −0.11, *p* = 0.009; Figure 5) with high statistical heterogeneity (I^2^ = 75%). Testing for subgroup differences showed statistical differences (*p* = 0.01) between the supervised and non-supervised short-term exercise programs (Figure 5). 

A sensitivity analysis omitting each study was performed and it did not affect the findings.

##### Long-Term Interventions (More Than 12 Weeks)

**Figure 6:**
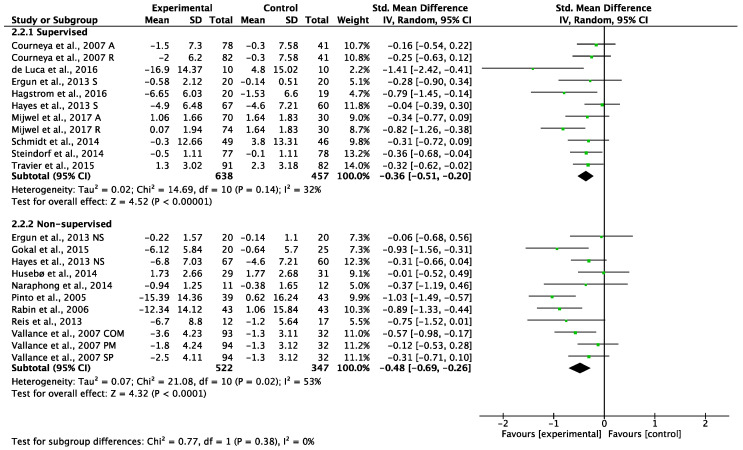
Meta-analyses of the effects of supervised [46,48,49,50,52,53,58,59,64] and non-supervised [38,40,42,49,57,59,60,62,65] exercise interventions of more than 12 weeks on CRF in BC patients. A: aerobic exercise group; COM: combination of printed materials and step pedometer; NS: non-supervised exercise group; PM: printed materials; R: resistance exercise group; S: supervised group; SP: step pedometer.

Long-term supervised exercise programs showed a significant reduction in CRF (SMD: −0.36, 95% CI −0.51 to −0.20, (*p* < 0.00001), I^2^ = 32%; Figure 6). Non-supervised exercise programs also showed a significant reduction in CRF (SMD: −0.48, 95% CI −0.69 to −0.26, (*p* < 0.0001), I^2^ = 53%; Figure 6). Testing for subgroup differences showed no statistical difference (*p* = 0.38) between the supervised and non-supervised long-term exercise programs (Figure 6). A sensitivity analysis omitting each study was performed and did not affect the findings.

#### 3.5.2. Subgroup Analysis: Adherence of the Training Programs

A subgroup analysis was performed to assess the impact of adherence to the exercise program on CRF. Studies with a lower level of adherence (≤80%) and higher level of adherence (>80%) were compared for both supervised and non-supervised programs.

##### Low Levels of Adherence (Less Than 80%)

**Figure 7:**
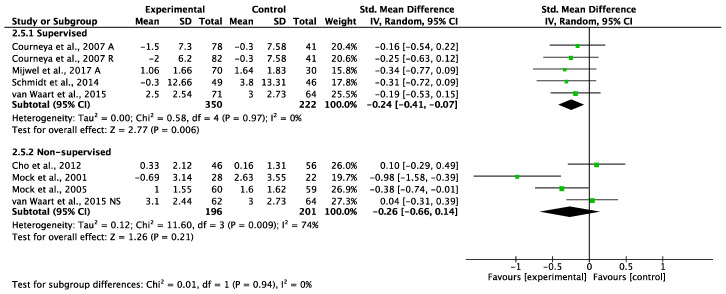
Meta-analyses of the effects of supervised [46,51,52,64] and non-supervised [36,37,41,51] exercise interventions of less than 80% adherence on CRF in BC patients. A: aerobic exercise group; NS: non-supervised exercise group; R: resistance exercise group.

Supervised exercise programs with lower levels of adherence showed a significant reduction in CRF (SMD: −0.24, 95% CI −0.41 to −0.07, *p* = 0.006, I^2^ = 0%; Figure 7). However, non-supervised exercise programs with lower levels of adherence did not achieve significant reductions in CRF (SMD: −0.26, 95% CI −0.66 to 0.14, *p* = 0.21; Figure 7) and had borderline high statistical heterogeneity (I^2^ = 74%). Testing for subgroup differences showed no statistical differences (*p* = 0.94) between supervised and non-supervised exercise programs with low levels of adherence (Figure 7). A sensitivity analysis omitting each study was performed and did not affect the findings.

##### High Levels of Adherence (More Than 80%)

**Figure 8:**
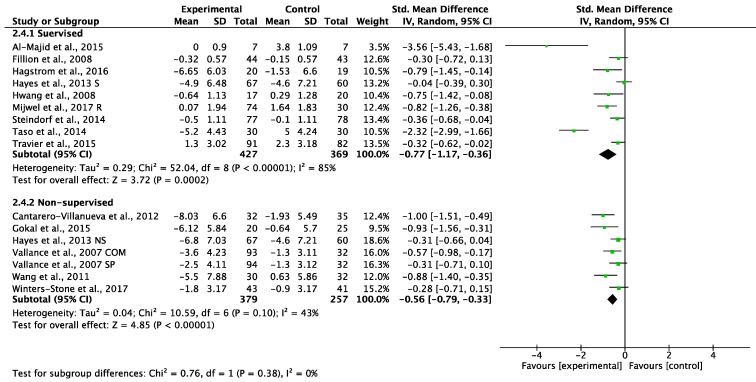
Meta-analyses of the effects of supervised [43,47,48,49,50,53,55,61,64] and non-supervised [39,45,49,54,57,65] exercise interventions of more than 80% adherence on CRF in BC patients. COM: combination of printed materials and step pedometer; NS: non-supervised exercise group; R: resistance exercise group; S: supervised group; SP: step pedometer.

Supervised exercise programs with higher levels of adherence showed a significant reduction in CRF (SMD: −0.77, 95% CI −1.17 to −0.36, (*p* = 0.0002); Figure 8) with high statistical heterogeneity (I^2^ = 85%). Non-supervised exercise programs with higher levels of adherence also showed a significant reduction in CRF (SMD: −0.56, 95% CI −0.79 to −0.33, (*p* < 0.00001), I^2^ = 43%; Figure 8). Testing for subgroup differences showed no statistical difference (*p* = 0.38) between supervised and non-supervised exercise programs with higher levels of adherence (Figure 8). A sensitivity analysis omitting each study was performed and it did not affect the findings.

## 4. Discussion

The primary aim of this systematic review and meta-analysis was to compare the effects of supervised and non-supervised exercise programs on CRF in women diagnosed with BC. Additionally, we aimed to determine the impact of intervention duration and adherence to the program on CRF in this patient group. The results of this meta-analysis show that both supervised and non-supervised exercise are beneficial in terms of reducing CRF severity in women with BC. This is consistent with previous studies, such as a meta-analysis by Meneses-Echávez et al., which included supervised interventions based on resistance, endurance, and stretching exercises, reporting significant reductions in CRF [79]. Similarly, the conclusions of Cheng et al., following their systematic review of non-supervised exercise programs, showed short-term benefits on symptom severity (i.e., fatigue, anxiety, and insomnia) and quality of life [80]. However, no previous studies have compared the relative benefits of supervised and non-supervised exercise for reducing CRF symptoms in women treated for BC.

Our results show that the difference in the magnitude of reduction in CRF symptoms between supervised and non-supervised interventions (SMD: −0.74 vs. −0.46, respectively) is statistically non-significant (*p* = 0.09) on the basis of current evidence (Figure 4). Supervised interventions lasting for <12 weeks were significantly more effective in reducing CRF than non-supervised exercise programs of similar duration (*p* = 0.002). Therefore, it seems that, in the short term, supervised exercise programs may be more effective than non-supervised programs for the management of CRF. These results suggest that exercise programs which include supervision can achieve good results in terms of CRF management in BC patients within a short timescale (i.e., within 12 weeks). However, as the duration of the exercise program increases (>12 weeks), the level of supervision seems less important. Interestingly, the effect size reduction in CRF for short-term supervised programs was notably higher than for non-supervised programs of longer and shorter durations and for supervised programs of a longer duration. This probably reflects the more intense level of supervision (and potentially higher adherence levels) in short-term programs, which may be difficult to maintain in the long term. Nevertheless, statistically significant reductions in CRF were still observed for supervised and non-supervised exercise programs of longer duration. This raises the question of whether the CRF-reducing effects of longer-term exercise programs could be improved by maintaining some level of supervision or by ensuring that short-term interventions develop the skills and confidence BC patients need for longer-term exercise behavior changes. 

Exercise adherence in this population appears to be influenced by prescription requirements and the level of supervision [81,82]. Previous studies suggest that exercise programs with high levels of supervision (one trainer per patient) and with just one session per week can increase adherence among BC patients [83]. In the current review, non-supervised training programs reported low levels of adherence. For example, four of the included studies achieved less than 80% adherence [36,37,41,51]. This low level of adherence may be attributable to the time period when the interventions took place (e.g., during BC treatments). In contrast, six studies achieved higher levels of adherence [39,45,49,54,57,65]. Among these studies, Cantarero-Villanueva et al. (2012), Hayes et al., (2013) and Vallance et al. (2007) may have achieved high levels of adherence because the exercise program was implemented after primary BC treatment, while higher adherence in the other three could be attributable to the provision of motivational tools. Gokal et al. (2015) provided a pedometer to measure daily step count, Wang et al. (2011) included heart rate (HR) monitoring during the intervention, and Winter-Stone et al. (2017) provided a cancer-specific yoga DVD. Pedometer step counts and HR monitoring provide immediate feedback and may encourage motivation [57,65]. Recently, smartphone apps and wearables have become increasingly available and are considered viable tools for collecting daily physical activity data and motivating behavior change [84]. Lynch et al. (2019) concluded that, with such technologies, it is possible to increase moderate to vigorous physical activity levels and reduce sedentary behaviors in BC survivors [85]. Therefore, technologies such as wearable devices may provide a great opportunity to monitor the intensity and duration of exercise and promote improved adherence in long-term interventions lasting >12 weeks. 

Aside from implementing steps to improve long-term adherence, there appears to be a dose–response relationship between exercise intensity and CRF, with the best results achieved at moderate-to-vigorous intensity [86]. Mijwel et al. (2019) suggest that high-intensity exercise can be a powerful strategy for helping BC patients to manage a range of health-related outcomes during chemotherapy [87]. According to the American College of Sport Medicine (ACSM), only one of the included studies [45] included resistance exercise reaching vigorous intensities (75 percent of one repetition maximum (%1RM)). In another study, participants were encouraged to perform exercise at moderate-to-vigorous intensity [65]; however, no specific devices (i.e., heart rate monitors) or scales (i.e., ratings of perceived exertion (RPE)) were used to verify the actual intensities achieved. In five studies [36,51,57,60,62], moderate intensity aerobic exercise was achieved using ratings of perceived exertion (RPE 12–14), and in another five studies [40,41,42,49,54] low-to-moderate intensity aerobic exercise was performed at 50–65 percent of maximum heart rate (%HRmax). A further study [39] engaged participants in low-intensity exercises and three studies [37,38,59] did not specify exercise intensity. This means that in just one of the included studies participants achieved moderate-to-vigorous intensity [45], which may be more effective for the management of CRF [86].

Age and stage of treatment are other factors which could potentially influence perceptions of CRF and, therefore, the responses to exercise therapy. However, the mean age of the patients included in the meta-analyses was very similar between supervised and non-supervised studies (51 years for supervised versus 53 years for non-supervised). Regarding the stage of treatment, a previous meta-analysis concluded that exercise during adjuvant therapy had no beneficial effect on CRF [88]. This is probably because CRF levels peak during chemotherapy [89] and women may be unable to effectively engage in exercise programs during adjuvant treatments. Consequently, it seems that the greatest impact of exercise on the management of CRF is observed when patients have completed their primary BC treatments. The proportion of participants that had completed their primary BC treatments when the exercise programs were implemented was similar in the supervised and non-supervised studies (43% and 55%, respectively). Thus, differences in the characteristics of participants and the stage of treatment appear to have had no effect on the observed effect size differences between supervised and non-supervised exercise programs.

This study has some limitations. Firstly, our analyses did not differentiate between the types of treatment patients were receiving or the stage of the disease. Both factors have the potential to influence how exercise impacts CRF in this patient group. Furthermore, although the average sample size for included studies was 91, 2 studies had less than 10 participants in the experimental group [35,43]. These low sample sizes can affect the likelihood that a statistically significant finding reflects a true effect [90]. Trials with small sample sizes may reduce the precision of the estimated effects and the spread of points in the funnel plot may widen [91], reaching asymmetry, as shown in Figure 3. If a small sample size had been an exclusion criteria, a greater symmetry in the funnel plot could have been achieved. Despite this, 26 of the included studies had >30 participants in their experimental group. Another limitation is that only 19 of the 31 included studies recorded adherence to the exercise program, representing just the 61% of the included studies. The heterogeneity of the sample is another factor to bear in mind when interpreting the results of the meta-analyses; for example, one study incorporated aquatic exercises [44], three included yoga lessons [35,39,55], one study evaluated the effectiveness of core stability exercises and myofascial release massage with endurance and resistance exercises [45], and another study involved ‘Nia exercises’, i.e., cardiovascular and whole-body-conditioning exercises [38]. Finally, many different questionnaires were used to assess CRF and this variety of assessment tools could have had some bearing on the results.

## 5. Conclusions

This is the first systematic review and meta-analysis to assess and compare the effects of both supervised and non-supervised exercise interventions for reducing CRF symptoms in women treated for BC. On the basis of current evidence, both supervised and non-supervised exercise programs are beneficial for reducing CRF severity in this patient group. However, a subgroup analysis of intervention duration showed statistical differences in favor of supervised interventions when the duration of the exercise program was <12 weeks. Further research is needed to understand how the CRF-reducing effects of long-term exercise programs could be improved in women recovering from BC treatment. Motivational strategies (e.g., wearable technologies), which provide the capability to monitor the intensity of exercise and promote greater adherence in the absence of intensive supervision, are important avenues for future research. 

## Supplementary Materials

The following supporting information can be downloaded at: https://www.mdpi.com/article/10.3390/cancers14143428/s1, Table S1: Study characteristics, File S1: PRISMA Checklist. References [35,36,37,38,39,40,41,42,43,44,45,46,47,48,49,50,51,52,53,54,55,56,57,58,59,60,61,63,64,65] are cited in the Supplementary Materials.

## Appendix A

Search strategy:

PubMed: ((“adult*”) AND (“breast*” OR “breast cancer” OR “breast tumors” OR “mammary cancer” OR “mammary carcinoma”) AND (“exercise” OR “acute exercise” OR “aerobic exercise” OR “exercise training” OR “exercise, aerobic” OR “exercise, isometric” OR “exercise, physical” OR “isometric exercise” OR “physical activity” OR “strength” OR “power” OR “endurance”) AND (“fatigue”)).

SportDiscus: TX (“exercise” OR “acute exercise” OR “aerobic exercise” OR “exercise training” OR “exercise, aerobic” OR “exercise, isometric” OR “exercise, physical” OR “isometric exercise” OR “physical activity” OR “strength” OR “power” OR “endurance”) AND TX (“breast*” OR “breast cancer” OR “breast tumors” OR “mammary cancer” OR “mammary carcinoma”) AND TX (“adult*”) AND TX (“fatigue”).

Web Of Science: ALL = ((“adult*”) AND (“breast*” OR “breast cancer” OR “breast tumors” OR “mammary cancer” OR “mammary carcinoma”) AND (“exercise” OR “acute exercise” OR “aerobic exercise” OR “exercise training” OR “exercise, aerobic” OR “exercise, isometric” OR “exercise, physical” OR “isometric exercise” OR “physical activity” OR “strength” OR “power” OR “endurance”) AND (“fatigue”)).

CINAHL: ((“adult*”) AND (“breast*” OR “breast cancer” OR “breast tumors” OR “mammary cancer” OR “mammary carcinoma”) AND (“exercise” OR “acute exercise” OR “aerobic exercise” OR “exercise training” OR “exercise, aerobic” OR “exercise, isometric” OR “exercise, physical” OR “isometric exercise” OR “physical activity” OR “strength” OR “power” OR “endurance”) AND (“fatigue”)).

PsycInfo: ((“adult*”) AND (“breast*” OR “breast cancer” OR “breast tumors” OR “mammary cancer” OR “mammary carcinoma”) AND (“exercise” OR “acute exercise” OR “aerobic exercise” OR “exercise training” OR “exercise, aerobic” OR “exercise, isometric” OR “exercise, physical” OR “isometric exercise” OR “physical activity” OR “strength” OR “power” OR “endurance”) AND (“fatigue”)).

CENTRAL: ((“adult*”) AND (“breast*” OR “breast cancer” OR “breast tumors” OR “mammary cancer” OR “mammary carcinoma”) AND (“exercise” OR “acute exercise” OR “aerobic exercise” OR “exercise training” OR “exercise, aerobic” OR “exercise, isometric” OR “exercise, physical” OR “isometric exercise” OR “physical activity” OR “strength” OR “power” OR “endurance”) AND (“fatigue”)).

ClinicalTrials.gov: ((“adult*”) AND (“breast*” OR “breast cancer” OR “breast tumors” OR “mammary cancer” OR “mammary carcinoma”) AND (“exercise” OR “acute exercise” OR “aerobic exercise” OR “exercise training” OR “exercise, aerobic” OR “exercise, isometric” OR “exercise, physical” OR “isometric exercise” OR “physical activity” OR “strength” OR “power” OR “endurance”) AND (“fatigue”)).

EMBASE: ((“adult*”) AND (“breast*” OR “breast cancer” OR “breast tumors” OR “mammary cancer” OR “mammary carcinoma”) AND (“exercise” OR “acute exercise” OR “aerobic exercise” OR “exercise training” OR “exercise, aerobic” OR “exercise, isometric” OR “exercise, physical” OR “isometric exercise” OR “physical activity” OR “strength” OR “power” OR “endurance”) AND (“fatigue”)).
