# Supplementary material for: Supervised and Non-Supervised Exercise Programs for the Management of Cancer-Related Fatigue in Women with Breast Cancer: A Systematic Review and Meta-Analysis
Source: Cancers (Basel). 2022 Jul 14;14(14):3428. doi: 10.3390/cancers14143428 (PMC9319207; doi:10.3390/cancers14143428)
Supplement: Supplementary file 1 [file cancers-14-03428-s001.zip › cancers-1769485-supplementary.pdf]

Supplementary Materials

Supervised and Non-Supervised Exercise Programs for the Management of Cancer-Related Fatigue in Women with Breast Cancer: A Systematic Review and Meta-Analysis.

Gonzalo Reverte-Pagola, Horacio Sánchez-Trigo, John Saxton and Borja Sañudo

Table S1. Study characteristics.

References	Age (Sd)	Sample Characteristics	*n*	Adherence	Questionnaire	Intervention Description
Al-Majid et al., 2015 [43]	E: 48 (10)<br>C: 53 (11)	Undergoing chemotherapy treatments.<br>Stage of disease: I-II	E: 7<br>C: 7	95-97	PFS	*Aerobic*:<br>Mode: 5 min warm-up, treadmill exercise and ended with a 5 min cooldown.<br>Intensity: 1º Week 40-50%HRR; 2º-3º Week 50-70%HRR; 4º-12º Week 70-80%HRR.<br>Session duration: 1º Week: 20min; 2º-3º Week: 30min; 4º-12º Week: 30-40min<br>Frequency: 9-12 weeks. 1º Week: 2 d/w; 2º-3º Week: 2-3 d/w; 4º-12º Week: 2-3 d/w.<br>Supervision: YES<br><br>*Control group:* Usual care.
Banasik et al., 2011 [35]	E: 63<br>C: 62	Completed treatments at least 2 months ago.<br>Stage of disease: II-IV	E: 7<br>C: 7	-	Quality Of Life Breast Cancer Specific Questionnaire	*Other exercise*:<br>Mode: Yoga. Traditional Iyengar yoga routines.<br>Intensity: progressively increased in difficulty as participants gained strength and flexibility.<br>Session duration: 90 min<br>Frequency: 8 weeks. 2 d/w.<br>Supervision: YES<br><br>*Control group:* Noninterventional control group.
Cantarero-Villanueva et al., 2012 [45]	E: 49<br>C: 48	Finished coadjuvant treatment except hormone therapy.<br>Stage of disease: I-IIIA<br>Treatment type: radiation (2), chemotherapy (6), radiation + chemotherapy (59).<br>Surgery: tumorectomy (42) or mastectomy (25).	E: 32<br>C: 35	80 - 87	POMS	*Combined*:<br>Mode: Aerobic, resistance and mobility exercise (core stability exercises; all major muscle groups; small soft ball, mats, fit-ball and resistance bands).<br>Intensity: Resistance exercise: 75% maximum load, 2–3 sets of 10–15 repetitions.<br>Session duration: 90 min<br>Frequency: 8 weeks. 3 d/w.<br>Supervision: NO<br><br>*Control group:* Usual health care.
Cantarero-Villanueva et al., 2013 [44]	E: 49 (7)<br>C: 47 (8)	Finished coadjuvant treatment except hormone therapy in the previous 18 months.<br>Stage of disease: I-IIIA<br>Treatment type: radiation (2), chemotherapy (6), radiation + chemotherapy (53).<br>Hormone therapy: tamoxifen (43), aromatase inhibitors (11), others (7).<br>Surgery: Lumpectomy (42) or Mastectomy (19).	E: 32<br>C: 29	-	PFS	*Other exercise*:<br>Mode: Hydrotherapy: 10 minutes of warm-up, 40 minutes of aerobic and endurance exercises, and 10 minutes of cool-down exercises.<br>Intensity: Moderate, 2–3 sets of 8–12 repetitions.<br>Session duration: 60 min.<br>Frequency: 8 weeks. 3 d/w.<br>Supervision: YES<br><br>*Control group:* Usual care.

Cho et al., 2012 [36]	51	Undergoing treatments and after cancer treatment.<br>Stage of disease: I-III	E: 46<br>C: 56	74	PFS	*Aerobic*:<br>Mode: Not specified.<br>Intensity: Moderate.<br>Session duration: 20 min.<br>Frequency: 9-12 weeks. 3 d/w.<br>Supervision: NO<br><br>*Control group:* Non-exercisers: defined as women who did not perform aerobic exercise of moderate intensity three times per week with a duration of 20 minutes per session.
Courneya et al., 2007 [46]	RE: 49<br>AE: 49	Undergoing treatments.<br>Stage of disease: I-IIIA.<br>Treatment type: Nontaxane chemotherapy protocol (167), Texane chemotherapy protocol (75).<br>Surgery: breast conservation (143).<br>Mean BMI: 26.6	RE: 82<br>AE: 78<br>C: 82	Resistance exercise group: 70<br><br>Aerobic exercise group: 70	FACT-F	*Resistance*:<br>Mode: 9 exercises (leg extension, leg curl, leg press, calf raises, chest press, seated row, triceps extension, biceps curls, and modified curl-ups). Warm-up and cool-down periods were 5 minutes of light aerobic activity and stretching.<br>Intensity: 2 sets of 8-12 repetitions at 60-70% 1RM. Resistance was increased by 10% when participants completed more than 12 repetitions.<br>Session duration: Not specified.<br>Frequency: 17 weeks. 3 d/w.<br>Supervision: YES<br><br>*Aerobic*:<br>Mode: Exercise on a cycle ergometer, treadmill, or elliptical. Warm-up and cool-down periods were 5 minutes of light aerobic activity and stretching.<br>Intensity: 1º-6º Week: 60% of their VO2max. 7º-12º Week: 70%. 12º-17º Week: 80%.<br>Session duration: 1º-3º Week: 15 min. Increased by 5 minutes every 3 weeks until the duration reached 45 minutes.<br>Frequency: 17 weeks. 3 d/w.<br>Supervision: YES<br><br>*Control group:* Usual care
Daley et al., 2004 [56]	18-65	Completed treatments within the past 12-36 months.	E: 33<br>C: 33	-	PFS	*Aerobic*:<br>Mode: Treadmill walking, cycling, rowing.<br>Intensity: 1º-4º Week: 60-75%. 5º-8º Week: 75-85%.<br>Session duration: 50 min (30 min exercise and 20 min exercise counselling)<br>Frequency: 8 weeks. 3 d/w.<br>Supervision: YES<br><br>*Control group:* Normal care.
de Luca et al., 2016 [58]	46 (3)	Completed treatments at least 6 months before.<br>Stage of disease: I-III.<br>Chemotherapy protocol (20), radiation therapy (12), hormonal therapy (10).<br>Mean BMI: E=24.6 and C=25.81.	E: 10<br>C: 10	-	FACT-F	*Combined*:<br>Mode: Aerobic training consisted of a stationary bike pedaling. Resistance training included five exercises for the main muscle groups performed on weight-lifting machines (leg curl; leg extension and leg press; shoulder press and vertical traction).<br>Intensity: Aerobic exercise: 70–80% HRmax; Resistance exercise: 2–4 sets per exercise, 6–10 repetitions at 40–60% 1RM.<br>Session duration: 90 min (10 min warm-up, 40 min resistance exercise, 30 min aerobic exercise, 10 min cool-down)<br>Frequency: 24 weeks. 2 d/w.<br>Supervision: YES

						Control group: Instructed not to begin any new formal physical exercise program during the study.
Ergun et al., 2013 [59]	E: 50 (8)<br>C: 50 (10)	Have completed treatments.<br>Surgery: partial mastectomy (25), total mastectomy (35).<br>Mean BMI: SG=26.55, NS=28.64, C=28.60.<br>Post-menopausal women.	SG: 20<br>NS: 20<br>C: 20	-	BFI	Supervised group:<br>*Combined*:<br>Mode: Aerobic: 30 min of brisk walking. Resistance: lower limb resistive exercises with Theraband set at moderate resistance and semi-squatting periods.<br>Intensity: Resistance: moderate resistance. Aerobic: not specified.<br>Session duration: 45 min of resistance exercise and 30 min of aerobic exercise.<br>Frequency: 12 weeks. 3 d/w resistance exercise and 3 d/w of aerobic exercise.<br>Supervision: YES<br><br>Non-supervised group:<br>*Aerobic*:<br>Mode: 30 min of brisk walking.<br>Intensity: Not specified.<br>Session duration: 30 min of aerobic exercise.<br>Frequency: 12 weeks<br>Supervision: NO<br><br>*Control group:* Education group.
Fillion et al., 2008 [47]	E: 54<br>C: 52	Had completed radiotherapy treatments.<br>Stage of disease: 0-III<br>Treatment type: chemotherapy (49), radiation therapy (87), hormonal therapy (64).<br>Surgery: lumpectomy (75), total mastectomy (12), axillary dissection (49).<br>Mean BMI: from <18.5 (3) to 35-39.9 (2).<br>Premenopausal (9), perimenopausal (44), postmenopausal (34).	E: 44<br>C: 43	96	MFI	*Aerobic*:<br>Mode: Walking training<br>Intensity: Not specified.<br>Session duration: 60 min + 90 min of psycho-educative.<br>Frequency: 4 weeks. 4 d/w.<br>Supervision: YES<br><br>*Control group:* Usual care.
Gokal et al., 2015 [57]	E: 52<br>C: 52	Undergoing chemotherapy.<br>Stage of disease: I-III<br>Treatment type: fluorouracil, epirubicin and cyclophosphamide or fluorouracil, epirubicin and cyclophosphamide followed by Taxotere.<br>Surgery: lumpectomy, mastectomy or segmental.<br>Mean BMI: E=27.20, C=28.25.<br>Pre- and post-menopausal.	E: 25<br>C: 25	80	FACT-F	*Aerobic*:<br>Mode: Walking.<br>Intensity: 12-14 RPE (6-20 scale)<br>Session duration: 10-30 min.<br>Frequency: 12 weeks. 5 d/w.<br>Supervision: NO<br><br>*Control group:* Usual care alone (medical care only).
Hagstrom et al., 2016 [48]	52 (9)	Completed surgery, radiotherapy, and/or chemotherapy, with or without hormonal therapy.<br>Stage of disease: I-IIIA<br>Treatment type: radiotherapy (34), chemotherapy (33), Tamoxifen or Arimidex (29).<br>Mean BMI: 28.7	E: 19<br>C: 15	85±15	FACT-F	*Resistance*:<br>Mode: 3 sets of 8-10 repetitions were performed of each exercise (leg extension, leg curl or Romanian deadlift, lat pull-down, machine bench press, seated row, back extension, prone hold or sit-ups)<br>Intensity: Loads were adjusted each time an individual had adapted to achieve the ability to complete 10 repetitions prior to muscular failure.<br>Session duration: 60 min.<br>Frequency: 16 weeks. 3 d/w.<br>Supervision: YES<br><br>*Control group:* Usual care.

Hayes et al., 2013 [49]	NS: 52<br>SG: 51<br>C: 54	3-4 weeks post-surgery.<br>Stage of disease: 0-III.<br>Treatment type: chemotherapy (117), radiotherapy (75), hormone therapy (114).<br>Surgery: lumpectomy (127), mastectomy (67).<br>Mean BMI: 26.6	NS: 67<br>SG: 67<br>C: 60	NS: 81<br>S: 88	FACT-F	*Combined*:<br>Mode: Incorporating both aerobic and strength-based exercises<br>Intensity: low–high.<br>Session duration: 20-45+ min.<br>Frequency: 32 weeks. ≥4 d/w.<br>Supervision: NO<br><br>*Control group:* Instructed not to begin any new formal physical exercise program during the study.
Husebø et al., 2014 [60]	E: 51<br>C: 54	Undergoing chemotherapy.<br>Stage of disease: I-III<br>Treatment type: Chemotherapy regimen of fluorouracil, epirubicin and cyclophosphamide 60; 100; 60 + Taxorete; 100 + Taxorete; 60 + Taxol. Radiotherapy (48).<br>Surgery: lumpectomy (45), mastectomy (22).<br>Mean Body weight: 70.5	E: 29<br>C: 31	-	Schwartz Cancer Fatigue Scale 6-30	*Combined*:<br>Mode: Endurance training: exercises with resistance bands for arms and legs. The aerobic prescription consisted of a daily 30 minutes of brisk walking.<br>Intensity: Moderate.<br>Session duration: Aerobic exercise: 30 mins. Resistance exercise: Not specified.<br>Frequency: 17 weeks. Aerobic: Daily. Resistance exercise: 3 d/w.<br>Supervision: NO<br><br>*Control group:* Regular physical activity level.
Hwang et al., 2008 [61]	E: 46 (8)<br>C: 46 (10)	Undergoing radiotherapy treatments.<br>Surgery: radical mastectomy (13), breast-conserving surgery with axillary lymph node dissection (12), breast-conserving surgery with sentinel lymph node biopsy (12)	E: 17<br>C: 20	100	BFI	*Combined*:<br>Mode: 10 min warm-up, 30 min of exercise (including stretching exercises focused on the shoulders, aerobic exercise such as treadmill walking and bicycling, and strengthening exercise), and a 10 min cool-down (relaxation period)<br>Intensity: Moderate. 50-70 HRmax.<br>Session duration: 50 min.<br>Frequency: 5 weeks. 3 d/w.<br>Supervision: YES<br><br>*Control group:* Self-shoulder stretching exercise.
Mijwel et al., 2017 [64]	AE: 54 (10)<br>RE: 53 (10)<br>C: 53 (10)	Undergoing treatments.<br>Stage of disease: I-IIIA.<br>Treatment: Anthracycline (80), Taxane (6), Anthracycline + Taxane (75), Anthracycline + Taxane + Herceptin (44), Anthracycline + Herceptin (2).<br>Premenopausal (84), postmenopausal (121).<br>Mean body mass: AE=67.7, RE=68.7, C=69.1.	AE: 70<br>RE: 74<br>C: 60	AE: 75<br>RE: 83	PFS	*Aerobic*:<br>Mode: Moderate-intensity aerobic and high-intensity interval training; 20 min of moderate intensity, continuous aerobic exercise at an RPE of 13–15 on a cycle ergometer, elliptical ergometer, or treadmill. Followed by 3 × 3min bouts of high intensity interval aerobic exercise on a cycle ergometer interspersed with one min of low-intensity active recovery.<br>Intensity: Aerobic: moderate intensity and then in the HIIT:16-18 on the Borg scale.<br>Session duration: 60 min.<br>Frequency: 16 weeks. 2 d/w.<br>Supervision: YES<br><br>*Combined*:<br>Mode: Resistance and high-intensity interval training. Endurance: 2-3 sets of 8-12 repetitions. Aerobic: 3 × 3 min bouts of high-intensity interval aerobic exercise on a cycle ergometer interspersed with one min of low-intensity active recovery.<br>Intensity: Endurance = initial intensity of 70% of 1RM progressing to 80% 1RM. Aerobic: 16-18 on the Borg scale.<br>Session duration: 60 min.<br>Frequency: 16 weeks. 2 d/w.<br>Supervision: YES<br><br>*Control group:* Usual care.

Mock et al., 2001 [37]	48	Undergoing treatments.<br>Stage of disease: I-IIIA<br>Treatment type: radiotherapy (32), chemotherapy (18).<br>Surgery: Lumpectomy (31), mastectomy (19).<br>Mean BMI: 25.66.	E: 28<br>C: 22	60	POMS	*Aerobic*:<br>Mode: Walking.<br>Intensity: Low–high walking.<br>Session duration: 10-15 min.<br>Frequency: 6 weeks. 5-6 d/w.<br>Supervision: NO<br><br>*Control group:* Usual care.
Mock et al., 2005 [41]	52 (9)	Undergoing chemotherapy (42) or radiation therapy (58).<br>Stage of disease: 0-IIIA<br>Mean BMI: 25.7	E: 54<br>C: 54	72	PFS	*Aerobic*:<br>Mode: Walking.<br>Intensity: 50-70% HRmax.<br>Session duration: 15-30 min.<br>Frequency: 6 weeks. 5-6 d/w.<br>Supervision: NO<br><br>*Control group:* Encouraged to maintain current levels of activity, but no exercise prescriptions or formal programs were offered.
Naraphong et al., 2014 [62]	47 (8)	Undergoing chemotherapy treatment.<br>Stage of disease: I-IIIA.<br>Treatment type: doxorubicin (adriamycin)/ cyclophosphamide (1), cyclophosphamide/ doxorubicin/ fluorouracil (11), cyclophosphamide/ methotrexate/ fluorouracil (1), fluorouracil/ epirubicin/ cyclophosphamide (1), docetaxel/ doxorubicin/ cyclophosphamide (3), doxorubicin (adriamycin)/ cyclophosphamide/ paclitaxel (4).<br>Surgery: modified radical mastectomy (19), other (4).	E: 11<br>C: 12	-	PFS	*Aerobic*:<br>Mode: Walking.<br>Intensity: 12-14 RPE (6-20 scale). 40-60% HRmax.<br>Session duration: 20-30min + 5 min warm-up + 5 min cool-down<br>Frequency: 12 weeks. 3-7 d/w.<br>Supervision: NO<br><br>*Control group:* Typical support and services provided through the cancer center.
Pinto et al., 2005 [40]	53 (10)	Treatment type: radiation (59), chemotherapy (48), hormone treatment (53).<br>Surgery: lumpectomy (19), lumpectomy with node dissection (43), mastectomy simple and node dissection (18), mastectomy with reconstruction (6).<br>Have completed treatments.<br>Stage of disease: 0-II.<br>Mean BMI: E=27.51, C=28.56.	E: 39<br>C: 43	-	Linear Analog Scale For Fatigue	*Aerobic*:<br>Mode: Brisk walking, biking, swimming or use of home exercise equipment.<br>Intensity: 55-65% HRmax<br>Session duration: 10-30 min.<br>Frequency: 12 weeks. 2-5 d/w.<br>Supervision: NO<br><br>*Control group:* Asked not to change their current level of activity during the 12 weeks.
Rabin et al., 2006 [42]	E: 53 (9)<br>C: 53 (10)	Have completed treatments.<br>Stage of disease: 0-II	E: 43<br>C: 43	-	10-Cm Linear Analog Scale 0-100	*Aerobic*:<br>Mode: Brisk walking.<br>Intensity: 55-65% HRmax.<br>Session duration: 10-30 min.<br>Frequency: 12 weeks. 2-5 d/w.<br>Supervision: NO<br><br>*Control group:* Asked not to change their current level of activity during the 12 weeks.
Reis et al., 2013 [38]	56 (11)	Have completed treatments.<br>Stage of disease: I-IIIC.	E: 12<br>C: 17	-	FACT-F	*Other exercise*:

		Treatment type: hormone therapy (26), chemotherapy (19).<br>Surgery: Lumpectomy (23), mastectomy (8), partial mastectomy (7), reconstruction (6), bilateral mastectomy (3).<br>Mean BMI: 29				Mode: Nia exercise: a cardiovascular and whole-body-conditioning program that integrates five sensations: strength, flexibility, mobility, agility, and stability.<br>Intensity: Not specified.<br>Session duration: 20-60 min.<br>Frequency: 12 weeks. 3 d/w.<br>Supervision: NO<br><br>*Control group:* Usual care.
Schmidt et al., 2014 [52]	53 (10)	Undergoing adjuvant chemotherapy.<br>Stage of disease: I-IV.<br>Treatment type: Herceptine therapy (26), Taxane-containing therapy (113), anthracycline therapy (168), hormone therapy (14).<br>Surgery: lumpectomy or mastectomy.<br>Mean BMI: 26	E: 49<br>C: 46	71	FAQ	*Resistance*:<br>Mode: 8 different machine-based exercises: 3 sets of 8-12 repetitions.<br>Intensity: 60-80% of 1RM.<br>Session duration: 60 min.<br>Frequency: 12 weeks. 2 d/w.<br>Supervision: YES<br><br>*Control group:* Relaxation control group.
Shobeiri et al., 2016 [63]	E: 43 (10)<br>C: 44 (9)	Completed treatments.<br>Stage of disease: I-II.<br>Treatment type: radiation therapy (24), chemotherapy (50), hormone replacement therapy (26).<br>Surgery: mastectomy (25), lumpectomy (28).<br>Mean BMI: E=27.68, C=27.21.	E: 30<br>C: 30	-	EORTC-QLQ-C30 Questionnaire	*Aerobic*:<br>Mode: Warm-up period followed by moderate-intensity aerobic exercises completed with a cool-down period. The aerobic exercises were moderate walking, stretching physical activity, and specific movements of arms and shoulders.<br>Intensity: 50-70% HRR.<br>Session duration: 40-60 min.<br>Frequency: 10 weeks. 2 d/w.<br>Supervision: YES<br><br>*Control group:* Told to maintain their sedentary lifestyle for 10 weeks.
Steindorf et al., 2014 [53]	56 (9)	Undergoing treatments.<br>Stage of disease: 0-III.<br>Pretreatment: neoadjuvant chemotherapy (30), surgery only (100), adjuvant chemotherapy (25).<br>Radiation technic: 3D (116), intensity-modulated radiation therapy (39).<br>Radiation boost (113).<br>Current trastuzumab therapy (11).<br>Current hormone therapy (75).<br>Surgery: lumpectomy or mastectomy.<br>Mean BMI: 27.2.	E: 77<br>C: 78	97	FAQ	*Resistance*:<br>Mode: 8 different machine-based resistance exercises: 3 sets, 8-12 repetitions.<br>Intensity: 60-80% 1RM.<br>Session duration: 60 min.<br>Frequency: 12 weeks. 2 d/w.<br>Supervision: YES<br><br>*Control group:* Progressive muscle relaxation without any aerobic or muscle strengthening components.
Taso et al., 2014 [55]	49 (10)	Undergoing treatments.<br>Stage of disease: I-III. Treatment type: Taxotere, doxorubicin (22), cyclophosphamide, doxorubicin and fluorouracil (15), cyclophosphamide, methotrex and fluorouracil (11), fluorouracil, epirubicin and cyclophosphamide (12).	E: 30<br>C: 30	90	BFI	*Other exercise*:<br>Mode: 10 min warm-up, 40 min Anusara yoga and gentle stretching and 10 min relaxation exercises.<br>Intensity: Not specified.<br>Session duration: 60 min.<br>Frequency: 8 weeks. 2 d/w.<br>Supervision: YES<br><br>*Control group:* Standard care and maintaining an ordinary daily activity routine.
Travier et al., 2015 [50]	E: 50 (8)<br>C: 50 (8)	Undergoing treatments.<br>Stage of disease: M0 (no distant metastasis).	E: 87<br>C: 77	83	MFI	*Combined*:

		Adjuvant treatment: chemotherapy (72), radiotherapy (62), none (70).<br>Mean BMI: E=25.8, C=26.6.<br>Premenopausal (115), postmenopausal (78).				Mode: 5 min warm-up, 25 min aerobic training, 25 min endurance training, 5 min cooling down. Aerobic training included interval training (3x2 min increasing to 2x7 min). Endurance training was performed for all major muscle groups (arms, legs, shoulder and trunk), 1-2 sets, 10-20 repetitions.<br>Intensity: Aerobic = at ventilatory threshold. Resistance = 45% 65% and 75% 1RM.<br>Session duration: 60 min.<br>Frequency: 18 weeks. 2 d/w.<br>Supervision: YES<br><br>*Control group:* Asked to maintain their habitual physical activity pattern up to week 18.
Vallance et al., 2007 [65]	58	Undergoing treatments.<br>Stage of disease: I-IIIA<br>Treatment type: surgery (377), chemotherapy (203), radiation (261), hormones (252).<br>Current hormone therapy: Tamoxifen (182), Aromatase inhibitor (42).<br>Mean BMI: 27.7.<br>Postmenopausal 232.	E: 84<br>C: 85	83	Fatigue scale from the FACT measurement system	*Aerobic*:<br>Mode: Not specified.<br>Intensity: moderate/vigorous.<br>Session duration: ≥30 min.<br>Frequency: 12 weeks. 5 d/w.<br>Supervision: NO<br><br>*Control group:* Standard public health recommendation for physical activity.
van Waart et al., 2015 [51]	51 (9)	Undergoing treatments.<br>Stage of disease: I-III	E: 62<br>C: 64	55	MFI	Non-supervised:<br>*Combined*:<br>Mode: combined resistance and aerobic.<br>Intensity: 12-14 RPE.<br>Session duration: >30 min.<br>Frequency: 9-12 weeks. 5 d/w.<br>Supervision: NO<br><br>Supervised:<br>*Combined*:<br>Mode: Aerobic = 30 min. Resistance = 6 large muscle groups were trained for 20 min with 2 sets of 8 repetitions.<br>Intensity: Aerobic = 50-80% of the maximal workload: 12-16 RPE. Resistance= 80% 1RM.<br>Session duration: 50 min.<br>Frequency: 9-12 weeks. 5 d/w.<br>Supervision: YES<br><br>*Control group:* Usual care.
Wang et al., 2011 [54]	50 (10)	Undergoing treatments.<br>Stage of disease: I-II.<br>Treatment type: Cyclophosphamide, anthracycline, and fluorouracil (9), cyclophosphamide, epirubicin, and fluorouracil 125 (28), cyclophosphamide, methotrexate, and fluorouracil (4), doxorubicin (Adriamycin), cyclophosphamide (Cytoxan), paclitaxel (Taxol) (31).<br>Radiation (32).<br>Surgery: breast-conserving treatment (30), modified radical mastectomy (36), simple mastectomy (6).	E: 30<br>C: 32	94	FACT-F	*Aerobic*:<br>Mode: Walking.<br>Intensity: 40-60% HRmax.<br>Session duration: 30 min.<br>Frequency: 6 weeks. 3-5 d/w.<br>Supervision: NO<br><br>*Control group:* Usual care.

		Reconstruction: yes (9), no (63).<br>Mean BMI: 22.45.<br>Postmenopausal (35), premenopausal (34), perimenopausal (3).				
Winters-Stone et al., 2017 [39]	E: 61 (11)<br>C: 55 (10)	Undergoing treatments or have completed treatments.<br>Stage of disease: 0-IV<br>Receipt of radiation therapy: past (39), current (4).<br>Receipt of chemotherapy: past (51), current (11).<br>Receipt of anti-estrogen therapy: past (45), current (36).	E: 43<br>C: 41	-	The Brief POMS	*Other exercise*:<br>Mode: Yoga program.<br>Intensity: Low intensity.<br>Session duration: 30 min.<br>Frequency: 8 weeks.<br>Supervision: NO<br><br>*Control group:* Oncologist verbal recommendation to exercise.

AE: aerobic exercise group; C: control group; D: day; E: experimental; SD: standard deviation; BFI: Brief Fatigue Inventory; EORTC QLQ: European Organization for Research and Treatment of Cancer Quality of Life Questionnaire; FACT-F: Functional Assessment of Cancer Therapy—Fatigue; FAQ: Fatigue Assessment Questionnaire; HIIT: high-intensity interval training; HRmax: maximum heart rate; HRR: heart rate reserve ; MFI: Multidimensional Fatigue Inventory; NS: non-supervised group; PFS: Piper Fatigue Scale; POMS: Profile of Mood State; RE: resistance exercise group; RM: repetition maximum; RPE: rating of perceived exertion; SG: supervised group; W: week.
